# *Varroa destructor* parasitism reduces hemocyte concentrations and prophenol oxidase gene expression in bees from two populations

**DOI:** 10.1007/s00436-018-5796-8

**Published:** 2018-02-12

**Authors:** Gun Koleoglu, Paul H. Goodwin, Mariana Reyes-Quintana, Mollah Md. Hamiduzzaman, Ernesto Guzman-Novoa

**Affiliations:** 1 School of Environmental Sciences, University of Guelph, Guelph, ON N1G 2W1 Canada; 2 Departamento de Medicina y Zootecnia en Abejas, FMVZ, Universidad Nacional Autónoma de México, 04960 Ciudad de Mexico, Mexico

**Keywords:** *Varroa destructor*, Honey bees, Parasitism, Cellular immunity, Hemocytes, Prophenol oxidase

## Abstract

Circulating hemocytes are responsible for defensive and healing mechanisms in the honey bee, *Apis mellifera*. Parasitism by the mite *Varroa destructor* and injection of *V. destructor* homogenate in buffer, but not buffer injection, showed similar reductions in total hemocyte concentrations in both Africanized and European adult honey bees. This indicated that compounds in *V. destructor* homogenate can have similar effects as *V. destructor* parasitism and that the response is not solely due to wounding. Samples from honey bees with different hemocyte concentrations were compared for the expression patterns of hemolectin (*AmHml*), prophenol oxidase (*AmPpo*), and class C scavenger receptor (*AmSRC-C*). Of the genes tested, only the expression of *AmPpo* correlated well with hemocyte counts for all the treatments, indicating that melanization is associated with those responses. Thus, the expression of *AmPpo* might be a suitable biomarker for hemocyte counts as part of cellular defenses against injection of buffer or mite compounds and *V. destructor* parasitism and perhaps other conditions involving healing and immunity.

## Introduction

Hemocytes in insects are blood cells that circulate in the plasma fluid inside the hemocele (Ribeiro and Brehelin 2006). Cellular responses are part of the innate immune system of insects and involve hemocytes in processes such as phagocytosis, nodulation, and encapsulation (Lavine and Strand 2002). In addition, hemocytes play a major role in wound healing with hemocyte recruitment followed by aggregation at the wound site and release of granular components and other active chemicals forming a clot-like matrix (Theopold et al. 2002; Iwanaga and Lee 2005). Hemocytes are produced during the larval stage of honey bees (*Apis mellifera*) in lymphatic glands located on either side of the aorta (Hoffman 1993). When honey bees mature and become adults, these glands disappear and hemocyte production stops. Hemocytes are then stored under the cuticle or attached to the viscera until needed, at which point they are rapidly recruited to increase their density at the location where required (e.g., a site of wounding or ingress of a parasite) (Richards and Edwards 2002).

The mite, *Varroa destructor*, is one of the most deleterious parasites of the honey bee (Anderson and Trueman 2000; Guzman-Novoa 2016). The mite feeds by breaking through the bee’s cuticle with its stylet and then sucking up hemolymph (Kanbar and Engels 2003; Evans 2006). Kanbar and Engels (2003) demonstrated that *V. destructor* is able to create and maintain open wounds in the honey bee cuticle and repeatedly feeds upon the same open wounds over a long period of time, suggesting that the normal healing of the wounds is in some way retarded by the mite. This could be due to components of the mite saliva, which are secreted through the stylet, as the addition of extracted *V. destructor* saliva to hemocytes of tomato moth larvae (*Lacanobia oleracea*) reduced their ability to form pseudopodia and aggregates (Richards et al. 2011). A negative effect on honey bee hemocyte responses to *V. destructor* parasitism was reported by Belaid and Doumandji (2010), who found that the total hemocyte counts in the hemolymph of honey bees parasitized by *V. destructor* were significantly lower than parasite-free bees from the same colonies.

The aim of this study was to compare the effect of *V. destructor* parasitism on hemocyte concentrations of honey bees relative to the effect of wounding (by injection of buffer) or introduction of mite compounds (by injection of mite homogenate). Both European and Africanized *A. mellifera* genotypes were studied, as Africanized bees have been reported to have greater resistance to *V. destructor* than European bees due to behavioral mechanisms (Moretto et al. 1991; Arechavaleta-Velasco and Guzman-Novoa 2001; Guzman-Novoa et al. 2012) as well as their brood being less attractive to *V. destructor* (Guzman-Novoa et al. 1996; Page and Guzman-Novoa 1997; Guzman-Novoa et al. 2011). A second aim of this study was to determine if the expression of the honey bee genes linked with hemocytes was affected similarly as hemocyte concentrations. The expression of several honey bee genes could be expected to change along with the number of hemocytes. For example, hemolectin genes encode a clotting factor, and its expression was specific to hemocytes and lymph glands in *Escherichia coli*-challenged *Drosophila melanogaster* (Irving et al. 2005). Another example is class C scavenger receptor genes that encode a pattern recognition protein for detection of microorganisms, and its expression was the only one among 22 genes examined that was strongly expressed solely among hemocytes in *Bombyx mori* (Nakahara et al. 2009). A third example is prophenol oxidase genes that encode an enzyme for melanization, and prophenol oxidase expression was higher in hemocytes compared to whole *D. melanogaster* larvae (Irving et al. 2005; Tsakas and Marmaras 2010). Thus, there could be a direct relationship between expression of one or more of these genes and total hemocyte counts under the conditions of this study.

## Materials and methods

### Source of honey bees, mites, and mite homogenate

European honey bees of the Buckfast strain were reared at the Honey Bee Research Center of the University of Guelph in Guelph, ON, Canada. Queen honey bees of this genotype were bred in isolation on Thorah Island, ON, Canada, to ensure the purity of the strain. Africanized honey bees were reared at the Center for Environmental Education in Xochimilco, Distrito Federal, Mexico. The populations of Africanized and European bees were assessed by morphometric (Sylvester and Rinderer 1987) and mitochondrial DNA (Nielsen et al. 2000) analyses. Eight honey bee source colonies of each genotype were selected and treated with fluvalinate strips (Apistan®, Novartis, Mississauga, ON, CA) for 6 weeks prior to the experiments to control *V. destructor* infestations. Very low (< 1%) levels of *V. destructor* infestation rates were confirmed prior to the experiments by determining infestation rates in adult workers (De Jong et al. 1982). To obtain adult bees, frames containing emerging brood were taken from source colonies and incubated overnight inside screened emergence cages (5 × 28 × 25.5 cm) at 32–35 °C and 60% RH. Newly emerged adults from these frames were collected and treated as described as follows.

To obtain *V. destructor* females, colonies with greater than 15 mites per 100 adults were selected to obtain the parasites as described by Hamiduzzaman et al. (2012). *V. destructor* that were used for artificial infestation of honey bees were starved for 6 h prior to use. To prepare homogenate, *V. destructor* were placed in PBS (0.038 M anhydrous monosodium phosphate, 0.162 M disodium phosphate, 0.75 M sodium chloride, pH 7.4) immediately after collection and then vortexed for 15 s. After this washing, the PBS was removed and approximately 100 mites were blotted dry and placed in a sterile mortar with 5 μL of PBS per mite. They were ground with a mortar and pestle until no visible particles of their exoskeleton remained. The resulting homogenate was centrifuged at 16,500 g for 10 min, and the supernatant was removed and stored at − 20 °C.

### Treatments

To obtain sufficient numbers of bees to assess expression of multiple genes and measure hemocytes, 64 to 70 honey bees were used for each treatment. Injections were performed using a 32-gauge syringe needle between the second and the third tergite. Buffer injection treatment used 2.5-μL PBS, which has previously been used as a control for injections of honey bees (Aronstein and Saldivar 2005; Randolt et al. 2008; Koleoglu et al. 2017). *V. destructor* homogenate injection treatment used 2.5 μL of homogenate in PBS. After treatment, adults were placed in a screened hoarding cage (12.7 × 8.5 × 14.5 cm). For *V. destructor* parasitism treatment, honey bees were placed in screened hoarding cages. Two mites were placed on each honey bee through the cage screen using a fine brush. Only honey bees with *V. destructor* still attached at the sampling times were used to ensure parasitism. Also, *V. destructor* were removed from the adults at sampling to check that they were viable by observing if they moved their legs when flipped upside down and probed with a paintbrush. Honey bees for all treatments were fed a 50% sucrose solution and water ad libitum and incubated at 32–35 °C and 60% RH for 48 h. As a control, non-treated adults were incubated similarly in hoarding cages. Samples of honey bees were collected at 0 h post treatment (hpt) (just before treatment) and at 2, 12, 24, and 48 hpt. The experiment was repeated three times.

### Hemocyte quantification

Immediately after collection, four honey bees per treatment at each time point were pierced with a #7 entomological needle (Bioquip, Rancho Dominguez, CA, USA) through the membrane between the second and the third dorsal tergites and gently squeezed to extract 4 μL of hemolymph, which were loaded on a micropipette and evenly spread over a 1-cm^2^ area on a microscopy slide that was then air dried for 15 min. The hemocytes were stained using a Giemsa stain analogue (Hema 3® Stat Pack kit: Fisher Scientific, Fair Lawn, NJ, USA), following the manufacturer’s instructions with the following modifications. In the first step, 95% methanol was applied on the fixed sample instead of the fixative agent included in the kit (> 99% methanol, < 1% fast green), and the second staining agent, Solution II, was diluted 4×, because non-diluted Solution II stained the samples too intensely to allow hemocyte counting. After staining, the hemocytes were counted with a Leica CME® light microscope (Fisher Scientific, Fair Lawn, NJ, USA) using an ocular reticule grid containing 100 cells, each of which was 2.5 μm^2^ at ×400 magnification. Twenty cells on the grid were counted in a zig-zag pattern. Bodies stained light purple (cytoplasm) with a dark stain in the center (nucleus) were counted as hemocytes and were differentiated from other structures (e.g., pollen grains) by morphology, size, and staining pattern.

The number of hemocytes per microliter of hemolymph in each sample was calculated by no. hemocytes/μL = (ave. no. hemocytes/8 areas) × (1322)/4 μL, where the average number of hemocytes counted in 8 areas delimited by an ocular reticule was multiplied by 1322 to convert that area into the area covered by hemolymph on the slide, and then divided by the 4 μL of hemolymph applied to the slide.

### RNA extraction, cDNA synthesis, and semi-quantitative relative RT-PCR

After hemolymph collection, honey bees were immediately frozen at − 70 °C. Total RNA from three bees per sample was extracted as per Chen et al. (2000). The extracted RNA was stored at − 70 °C. Three replicates of the treatments for RNA extraction were conducted. cDNA was prepared using a RevertAid™ H Minus First Strand cDNA Synthesis Kit (Fermentas, Burlington, ON, CA) following the manufacturer’s instructions, and the cDNA was stored at − 20 °C.

To design primers sequences for the honey bee homologs of hemolectin (*AmHml*), class C scavenger receptor (*AmSCR-C*), and prophenol oxidase (*AmPpo*), the NCBI nr/nt database limited to *A. mellifera* (tax id: 7460) was searched using BLASTp with the amino acid sequences of *D. melanogaster* hemolectin (GenBank NM_079336), *B. mori* class C scavenger receptor (GenBank AB436164), and *D. melanogaster* prophenol oxidase (GenBank NM_057464). Primers were designed and assessed with the GeneRunner software (Hastings Software, Hastings, NY). The primers were ordered from the Laboratory Services of the University of Guelph (Guelph, ON, CA).

PCRs were run in an Eppendorf AG 22331 Master Cycler (Eppendorf, Hamburg, DE). Each reaction contained 2 μL of cDNA, 5 units (1 μL *Taq* DNA polymerase (New England Biolabs, Pickering, ON, CA), 10× *Taq* reaction buffer, 1 μL 10 mM dNTPs, and 1 μM of each primer for both the target gene and the constitutive housekeeping gene, ribosomal protein S5 (RPS5) (Table 1) (Evans 2006). RPS5 has been previously used as constitutive housekeeping gene in honey bee larvae and adults to normalize gene expression and was chosen based on macro-array analyses (Evans 2004). Reaction conditions were 35 cycles of 30 s at 94 °C, 60 s at 58 °C and 60 s at 72 °C, and a final extension step at 72 °C for 10 min. PCR products were separated on a 1% TAE agarose gel with 1% ethidium bromide and visualized using a BioDoc-It™ Imaging System (UVP, Mississauga, ON, CA) under UV light.

**Table 1:** Primers for amplification of the genes used in this study

Target gene description	Target gene	Forward (F) and reverse (R) primers	Product length	Source
Hemolectin	*AmHml*	F: 5’-CTGAAGACTTTACTGGACCAC-3’ R: 5’-TCGATGGAAGACAATTACC-3’	273 bp	This study
Prophenol oxidase	*AmPpo*	F: 5’-GTTAGACTTCCCAGGTATCG-3’ R: 5’-CACGCTCGTCTTCTTTAGG-3’	260 bp	This study
Scavenger class C protein	*AmSCR-C*	F: 5’-TTACGACGGTTTCACTCTTGC-3’ R: 5’-CAACGTGTATCCAGGCTCG-3’	184 bp	This study
Ribosomal protein S5	*RPS5*	F: 5’-AATTATTTGGTCGCTGGAATTG-3’ R: 5’-TAACGTCCAGCAGAATGTGGTA-3’	115 bp	Evans (2006)

The number of pixels of the bands for the target and constitutive control genes in each lane of the gel pictures were quantified using the Scion Image software (Scion Corporation, Frederick, MD, USA) as per Dean et al. (2002). To calculate relative expression, the intensity value for the target gene was divided by that of the constitutive gene. Each value was calculated from six measurements of three replications of the treatments with two technical repetitions of the PCR conditions. To determine that the number of cycles was not too great to determine relative expression, gene expression from randomly selected samples was also tested with five fewer cycles, and the patterns of expression were confirmed to be similar to those obtained using 35 cycles.

### Statistical analyses

The link between gene expression and hemocyte counts was examined running a linear regression analysis using Microsoft Excel. The data were adjusted to total hemocyte counts/microliter of hemolymph and were natural log (ln) transformed, because they were not normally distributed. The data were subjected to ANOVA and linear regression using IBM-SPSS v. 23 (SPSS Inc., Chicago, IL, USA).

## Results

### Hemocyte quantification

**Fig. 1:**
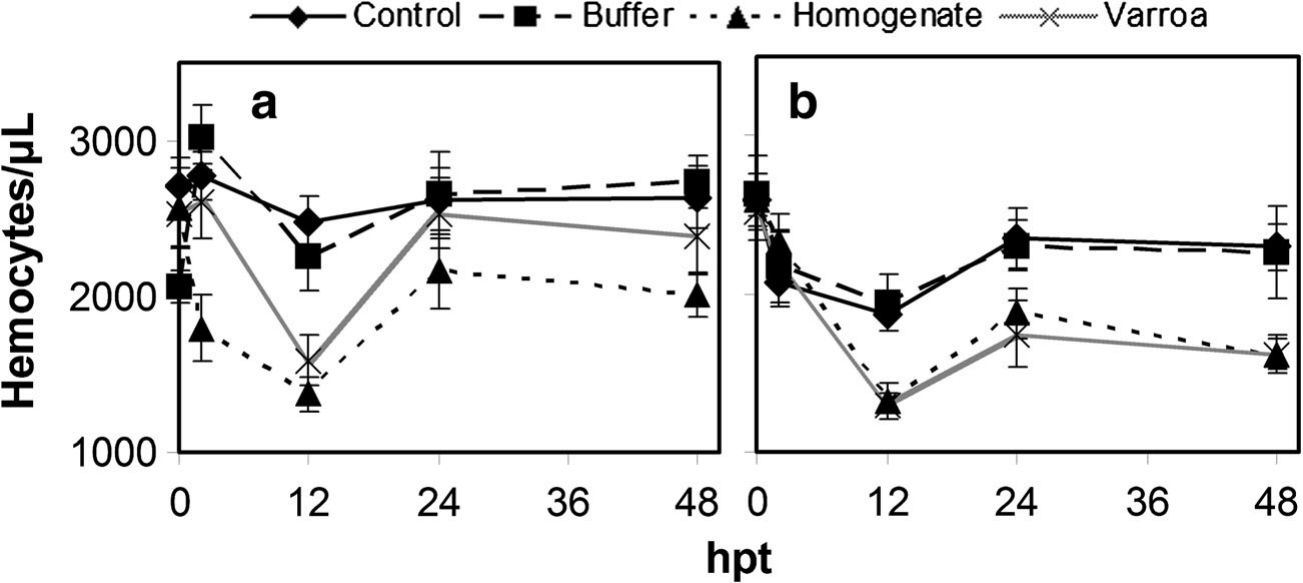
Hemocyte concentrations in European (**a**) and Africanized (**b**) adult honey bees in response to caging, buffer injection, *V. destructor* homogenate injection, and *V. destructor* parasitism. The treatments are cage control (solid line), buffer injection (dashed lines), *V. destructor* homogenate injection (dotted line), and *V. destructor* parasitism (gray line). Data points represent the mean number of hemocytes per microliter of hemolymph in 12 honey bees from three treatment replications with error bars representing standard error

Hemocyte concentration showed no significant changes over time for the European bee control (*F*_(4, 111)_ = 0.36; *p* = 0.837) (Fig. 1a). However, the hemocyte concentration for the Africanized bee control at 2 and 12 hpt was significantly different from 0 hpt but then returned to levels similar to 0 hpt (*F*_(4, 310)_ = 3.973; *p* = 0.004) (Fig. 1b). Thus, Africanized bees initially may have been somewhat more affected by caging than European bees, but the effect was temporary. At 2 hpt, none of the treatments were significantly different to each other for European bees, except for the homogenate being significantly lower (*F*_(3, 55)_ = 6.08; *p* = 0.001). Also, none of the treatments affected hemocyte concentrations in Africanized bees at 2 hpt (*F*_(3, 146)_ = 0.294; *p* = 0.830). At 12 hpt, hemocyte concentrations for the control and buffer injection treatments were not significantly different from each other but were significantly higher than both homogenate and *V. destructor* parasitism, which were not significantly different from each other, for both European (*F*_(3, 60)_ = 11.755; *p* < 0.0001) and Africanized bees (*F*_(3, 151)_ = 7.553; *p* < 0.0001). Thus, buffer injection resembled the control, while the effect of homogenate injection and *V. destructor* parasitism resembled each other. At 24 hpt, there were no significant differences among the treatments for either European (*F*_(3, 48)_ = 0.494; *p* = 0.688) or Africanized bees (*F*_(3, 148)_ = 2.305; *p* = 0.079). At 48 hpt, European bees still showed no significant differences between the treatments (*F*_(3, 43)_ = 1.30; *p* = 0.286), but Africanized bees now showed that homogenate and *V. destructor* parasitism had significantly lower hemocyte concentrations than the control but not buffer injection (*F*_(3, 145)_ = 4.311; *p* = 0.006). In general, the patterns of hemocyte concentration over time with buffer injection closely resembled its control, while the patterns with *V. destructor* homogenate injection and *V. destructor* parasitism differed more from the controls and resembled each other for both genotypes, which was most obvious at 12 hpt when hemocyte concentrations were at their minimums.

### Link between gene expression and hemocyte concentration

**Fig. 2:**
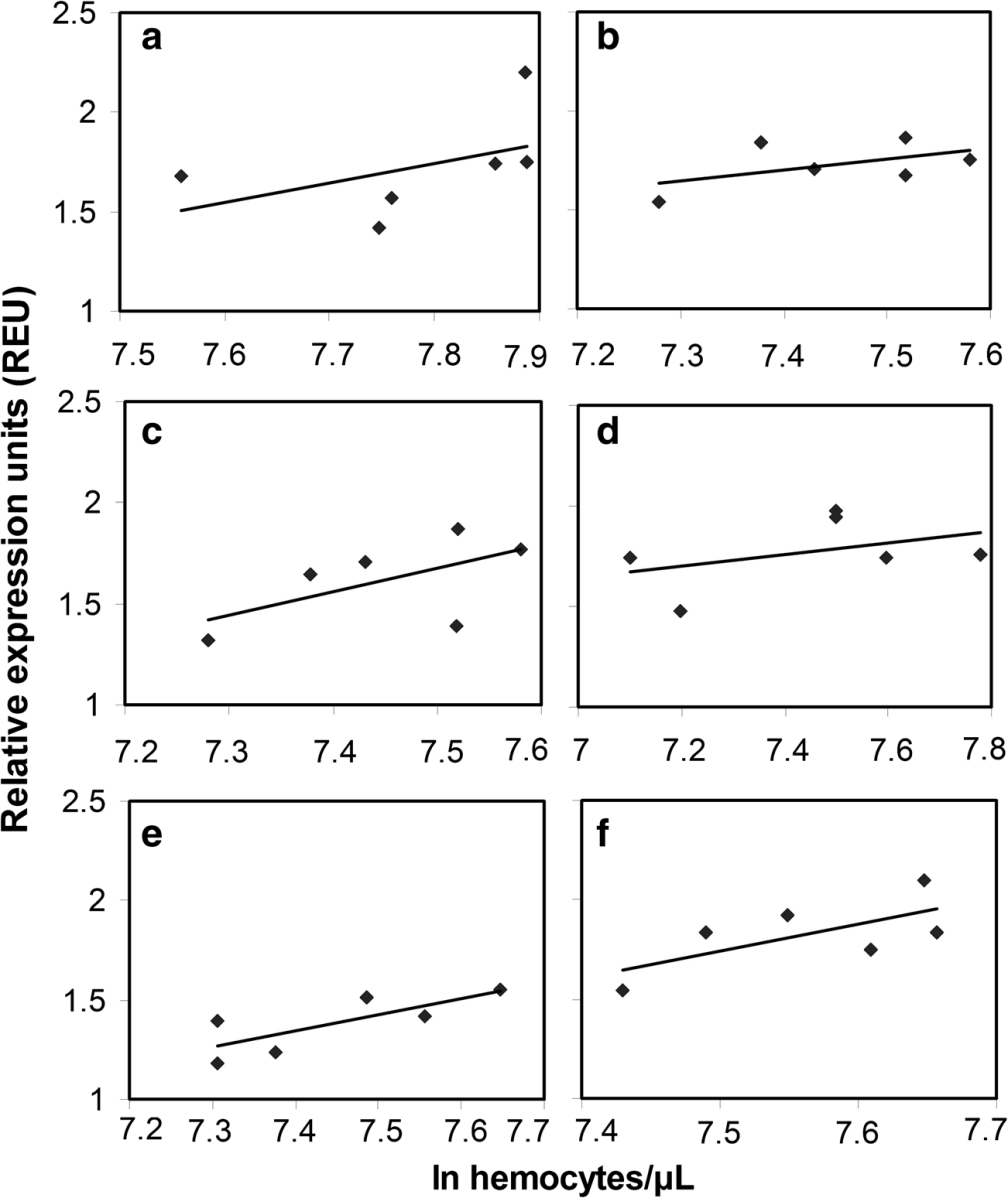
Regression analyses between *AmHml*, *AmSCR-C*, and *AmPpo* expression and ln hemocyte concentration in European and Africanized honey bees. *AmHml* expression in European bees (**a**), *AmHml* expression in Africanized bees (**b**), *AmSCR-C* expression in European bees (**c**), *AmSCR-C* expression in Africanized bees (**d**), *AmPpo* expression in European bees (**e**), and *AmPpo* expression in Africanized bees (**f**). Data from 0, 2, 12, 24, and 48 hpt were used for each genotype

The expression of *AmHml*, *AmSCR-C*, and *AmPpo* was determined for treatment samples from both European and Africanized honey bees with a range of hemocyte concentrations (Fig. 2). The primers for *AmHml* produced a single clear band of the predicted size of 273 bp, and regression analysis between the relative *AmHml* expression and hemocyte concentration showed low *R*^2^ values of 0.2492 (*p* = 0.47) and 0.2246 (*p* = 0.19) for Africanized and European bees, respectively. The primers for *AmSCR-C* produced a single clear band of the predicted size of 184 bp, and regression analysis between the relative *AmSCR-C* expression and hemocyte concentration revealed low *R*^2^ values of 0.1705 (*p* = 0.17) and 0.3329 (*p* = 0.33) for Africanized and European bees, respectively. The primers for *AmPpo* produced a clear single band of the predicted size of 260 bp. A regression between relative *AmPpo* expression and hemocyte concentration gave comparatively high *R*^2^ values of 0.4492 (*p* = 0.038) and 0.6033 (*p* = 0.021) for Africanized and European bees, respectively, indicating that the expression of *AmPpo* and hemocyte concentration correlated much better than *AmHml* or *AmSCR-C*.

### *AmPpo* expression

**Fig. 3:**
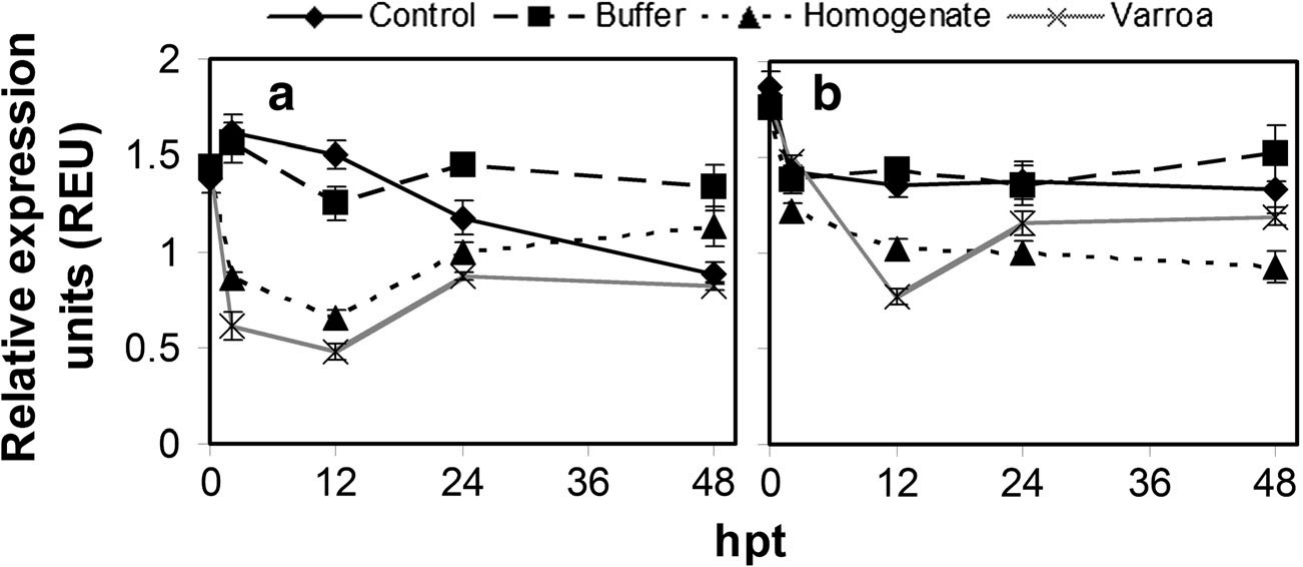
Relative expression of *AmPpo* in European (**a**) and Africanized (**b**) adult honey bees in response to caging, buffer injection, *V. destructor* homogenate injection, and *V. destructor* parasitism. The treatments are cage control (solid line), buffer injection (dashed lines), *V. destructor* homogenate injection (dotted line), and *V. destructor* parasitism (gray line). Data points represent the mean expression of nine honey bees from three treatment replications with two technical repetitions each and with error bars representing standard error

*AmPpo* expression was relatively stable for the European bee control until 12 hpt, but then decreased over time becoming significantly different from 0 hpt at 48 hpt (*F*_(4, 25)_ = 14.194; *p* < 0.0001) (Fig. 3a). For the Africanized bee control, there was a significant decline at 2 hpt but then expression remained relatively unchanged (*F*_(4, 25)_ = 5.25; *p* = 0.003) (Fig. 3b). Thus, unlike the changes in hemocyte concentration, changes in *AmPpo* expression indicate that European bees may be affected in the long term by caging. However, the effect on Africanized bees was temporary for both parameters. At 2 hpt, expression did not differ between control and buffer, but was significantly lower with homogenate and *V. destructor* parasitism than other treatments for European bees (*F*_(3, 20)_ = 39.4; *p* < 0.0001), but none of the treatments were significantly different from the control for Africanized bees except lower expression in homogenate injection than *V. destructor* parasitism (*F*_(3, 20)_ = 3.329; *p* = 0.040). At 12 hpt, *AmPpo* expression in European bees was significantly lower in homogenate injection and *V. destructor* parasitism, which were not different from each other, compared to the control and buffer injection (*F*_(3, 20)_ = 58.291; *p* < 0.0001). For Africanized bees, expression was also significantly lower in homogenate injection and *V. destructor* parasitism than the control and buffer injection, but *V. destructor* parasitism treatment was significantly lower than the homogenate treatment (*F*_(3, 20)_ = 32.57; *p* < 0.0001). At 24 hpt, the expression with homogenate injection and *V. destructor* parasitism remained significantly lower than control or buffer injected European bees (*F*_(3, 20)_ = 17.243; *p* < 0.0001). In Africanized bees at 24 hpt, *AmPpo* expression with homogenate injection was significantly lower than the control or buffer injection but not different from *V. destructor* parasitism (*F*_(3, 20)_ = 4.45; *p* = 0.015). At 48 hpt, the only significant difference in European bees was lower expression with *V. destructor* parasitism compared to buffer injection (*F*_(3, 20)_ = 7.90; *p* = 0.001), and the only significant difference in Africanized bees was the lower expression with homogenate compared to buffer injection (*F*_(3, 20)_ = 5.31; *p* = 0.007).

### Correlation of hemocyte concentrations and *AmPpo* expression

**Fig. 4:**
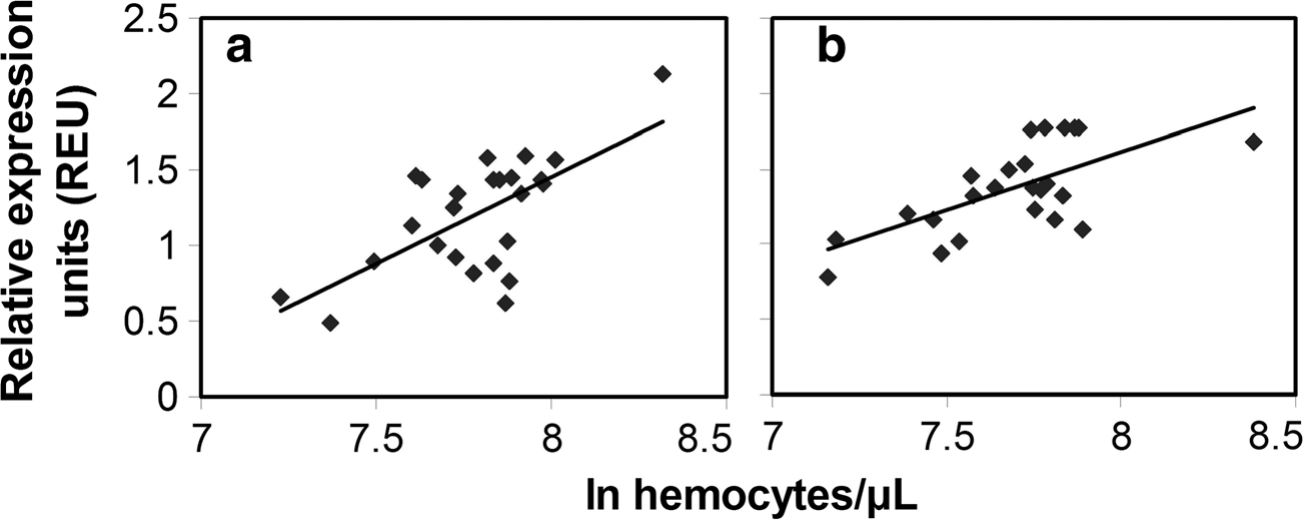
Regression analysis between *AmPpo* expression and ln hemocyte concentration of European (**a**) and Africanized (**b**) honey bees. Data from 0, 2, 12, 24, and 48 hpt were used for each genotype

A comparison between the hemocyte concentration and the *AmPpo* expression patterns showed several similarities indicating that they may be correlated (Figs. 1 and 3). A regression analysis between hemocyte concentrations and *AmPpo* expression levels showed *R*^2^ values of 0.4266 for European (*p* < 0.001) and 0.4404 for Africanized (*p* = 0.015) bees, showing that *AmPpo* expression was significantly related to hemocyte concentration under the controlled conditions of these experiments (Fig. 4).

## Discussion

Hemocytes mediate the four main insect cellular immune responses, which are phagocytosis, nodulation, encapsulation, and cytotoxicity (Zakaria 2007). Sessile clusters of hemocytes are stored in cuticular layers during larval development and then are recruited when needed during the remainder of the honey bee’s lifetime (Bahadur 1993). Their concentrations in the hemolymph can be affected by a number of factors, such as infections and abiotic stresses, and are related to other effects on honey bee physiology. For example, there were significant decreases in total hemocyte concentrations following infection with the bacterial pathogen, *Paenibacillus larvae* (Zakaria 2007), and exposure to sublethal doses of neonicotinoids decreased hemocyte concentration, encapsulation response, and antimicrobial activity of the hemolymph (Brandt et al. 2016).

For both the control and the buffer injection samples, hemocyte concentrations remained relatively unchanged over time for European and Africanized bees, except for a temporary decrease in Africanized bees. There was little difference in hemocyte concentrations between the control and the buffer injection treatments, indicating that wounding due to piercing the cuticle and buffer entering the hemolymph had no significant effect on hemocyte numbers over the course of the experiment.

Injection of *V. destructor* homogenate and *V. destructor* parasitism of honey bees caused very similar changes in hemocyte concentrations for European and Africanized bees. For both treatments, there was a decrease in hemocyte concentrations reaching a minimum at 12 hpt in both genotypes, which was greater than with the control of buffer injection treatment. Hemocyte concentrations with homogenate and *V. destructor* parasitism were not significantly different from each other at any time point, except at 2 hpt when concentrations with homogenate had declined faster than with *V. destructor* parasitism. This indicates that there are likely components in the homogenate and secreted during *V. destructor* parasitism that negatively affect this part of the immune system, which did not occur with only caging or wounding.

Richards et al. (2011) found that *V. destructor* saliva, which is secreted through the stylet during parasitism, contained multiple proteins, and the extracted saliva caused hemocytes to disintegrate leaving only debris. Thus, it is possible that the drop in hemocyte concentrations was due to some being destroyed by *V. destructor* saliva or components of the homogenate. Another possibility is that the *V. destructor* homogenate and saliva are interfering with the immune response in some manner, such as by degrading or preventing the detection of chemical cues from the wound site (Moreira et al. 2010). Little is known about the salivary secretions of *V. destructor*, but several *Ixodes* ticks, such as the deer tick *Ixodes scapularis*, have saliva containing a wide variety of factors including immunosuppressants, anti-inflammatory factors, anti-coagulants, anti-platelet aggregation factors, and vasodilators (Nuttall and Labuda 2004; Valenzuela 2004). Similar types of compounds may be present in *V. destructor*. These may have been extracted in the *V. destructor* homogenate, although the homogenate would also contain buffer soluble cytoplasmic material from mite cells, intestinal material, and symbionts that also could be affecting hemocyte concentrations.

There are several reports of gene expression correlating with hemocytes in insects. Irving et al. (2005) compared gene expression in extracted hemocytes to whole larvae of *D. melanogaster* and found 18 genes with higher expression in the hemocytes, including hemolectin and prophenol oxidase-2. A comparison of gene expression in extracted hemocytes to whole adults in *B. mori* identified 22 genes with higher expression in the hemocytes, such as class C scavenger receptor and prophenol oxidase (Nakahara et al. 2009).

In this study, a homolog of the *D. melanogaster* hemolectin gene was found for *A. mellifera*, but there was a poor correlation between expression of *AmHml* and hemocyte concentration. This was surprising as hemolectin acts as a clotting factor in hemolymph of insects (Goto et al. 2001), and the expression of hemolectin was specific to hemocytes and lymph glands in microbially challenged *D. melanogaster* (Irving et al. 2005). Hemolectin genes are expressed in plasmatocytes but not lamellocytes in *D. melanogaster* (Goto et al. 2003). Possible reasons for the poor correlation with hemocyte concentration could be that hemolectin expression had a treatment-specific response unrelated to hemocyte concentration or that only certain types of hemocytes were altered by the treatments.

Although a homolog of the *B. mori* class C scavenger receptor gene was found in *A. mellifera*, expression of *AmSCR-C* did not correlate with hemocyte concentrations. Class C scavenger receptors are conserved macrophage bound recognition proteins (Hampton et al. 1991; Ramet et al. 2001). In *B. mori*, class C scavenger receptor expression was much more strongly expressed in hemocytes than in the rest of the insect body (Nakahara et al. 2009). Its expression was restricted to hemocytes during embryonic development in *D. melanogaster* (Pearson et al. 1995). Like hemolectin, a lack of correlation of class C scavenger receptor gene expression with total hemocyte concentrations could be due to changes in expression due to treatment-specific responses or that only certain types of hemocytes were altered by the treatments.

In contrast to *AmHml* and *AmSCR-C*, *AmPpo* expression showed a positive correlation with hemocyte concentration for both bee genotypes, although the correlation was stronger in Africanized (*p* < 0.001) than in European bees (*p* = 0.015). Prophenol oxidase catalyzes the synthesis of melanin, which can contribute to the immune response (Cerenius and Soderhall 2008; Kanost and Gorman 2008). The expression of prophenol oxidase genes in *D. melanogaster* is induced by hemocyte death during the coagulation of hemolymph due to stress and recognition of injury chemical cues (Bidla et al. 2007). Three prophenol oxidase genes are found in *D. melanogaster*, and all have high levels of expression in hemocytes and very low levels of expression in other tissues (Irving et al. 2005). In honey bees, however, only one copy of a prophenol oxidase gene is present (Lourenco et al. 2004). *AmPpo* was most similar to prophenol oxidases 2 and 3 of *D. melanogaster* based on the alignment of amino acid sequences. The significant correlation between *AmPpo* expression and hemocyte concentrations for both honey bee genotypes was likely due to the relationship of *AmPpo* expression to melanization, which in turn is closely connected to clotting (Laughton et al. 2011).

Considering the good correlation between *AmPpo* expression and hemocyte concentrations, it was not surprising that the pattern of *AmPpo* expression and hemocyte concentrations were very similar over the 48 h following caging, buffer injection, homogenate injection, or *V. destructor* parasitism. Both *AmPpo* expression and hemocyte concentrations were most similar between caging and buffer injection but different from homogenate injection and *V. destructor* parasitism, which were most similar to each other. This was readily observable for hemocyte concentrations in Africanized bees. One notable difference between the two parameters was that *AmPpo* expression was more affected over time with the cage control than hemocyte concentrations in European bees. Another notable difference was that homogenate injection did not result in a notable minimum in *AmPpo* expression at 12 hpt in Africanized bees, unlike for hemocyte concentrations.

In general, the two honey bee genotypes responded similarly to the treatments for both *AmPpo* expression and hemocyte concentrations showing that no clear evidence that Africanized bees are more resistant to *V. destructor* based on the factors measured in this study under controlled conditions. The two honey bee genotypes also had a similar relationship between *AmPpo* expression and hemocyte concentrations. However, this study looked at a small number of factors, and future studies may find significant cellular and molecular differences between these genotypes in relation to *V. destructor*. The relationship between hemocyte concentrations and *AmPpo* expression in both genotypes shows that *AmPpo* expression could have broad applications as an bio-indicator of hemocyte concentrations.
